# DIVERSITY IN HOSPICE AND END-OF-LIFE EXPERIENCES: THE INFLUENCE OF CHRONIC DISEASE AND SOCIOCULTURAL FACTORS

**DOI:** 10.1093/geroni/igz038.2909

**Published:** 2019-11-08

**Authors:** David Russell, Elizabeth A Luth, Ruth Masterson Creber

**Affiliations:** 1 Visiting Nurse Service of New York, New York, New York, United States; 2 Weill Cornell Medicine, New York, New York, United States; 3 Weill Cornell Medical College, New York, New York, United States

## Abstract

Hospice provides supportive and palliative services to persons nearing the end-of-life. Use of the Medicare hospice benefit has grown to cover nearly half of all Medicare decedents. Even more notably, hospice agencies now serve patients with a diverse range of terminal conditions, including those not traditionally served by hospices, such as dementia and heart failure. In addition to expanded use of hospice care by patients with multiple types of chronic disease, demographic transitions in the United States over the last several decades have also led to increased use of hospice services among patients with diverse socio-cultural and linguistic backgrounds. Limited research has identified the unique experiences of patients enrolled in hospice who have diagnoses of dementia and heart failure, or explored how socio-cultural factors act to influence the course and outcomes of hospice care. This symposium features interdisciplinary collaborations between academic researchers and clinical practitioners at a large non-profit hospice agency in a multicultural urban environment. These collaborations, which draw on multiple theoretical perspectives and research methodologies, shed new light on patient experiences in hospice and identify opportunities for improving care and comfort at end-of-life. Presentations will include an exploration of the unique symptoms and experiences of hospice patients with heart failure, an evaluation of a clinical program for heart failure hospice patients, an exploration of collaborative goal setting between patients-providers, and an examination of cultural health capital as it relates to race/ethnic and socioeconomic disparities in hospitalization among hospice patients, and factors for disenrollment among hospice patients with dementia.

